# Vitamin D deficiency in low-birth-weight infants in Uganda; a cross sectional study

**DOI:** 10.1371/journal.pone.0276182

**Published:** 2022-11-11

**Authors:** Martin Chebet, Thereza Piloya, Faith Ameda, David Mukunya, Sarah Kiguli

**Affiliations:** 1 Department of Paediatrics and Child Health, Makerere College of Health Sciences, Kampala, Uganda; 2 Department of Paediatrics and Child Health, Busitema University Faculty of Health Sciences, Mbale, Uganda; 3 Department of Radiology, Makerere College of Health Sciences, Kampala, Uganda; 4 Department of Community and Public Health, Busitema University Faculty of Health Sciences, Mbale, Uganda; Public Library of Science, UNITED KINGDOM

## Abstract

**Background:**

Vitamin D deficiency affects 7–86% of infants globally and results in recurrent infections, impaired growth and nutritional rickets. Low-birth-weight infants in Uganda are at risk of vitamin D deficiency due to limited sunlight exposure and dependence on breastmilk. We aimed to determine the prevalence and factors associated with vitamin D deficiency among low-birth-weight infants aged 6 weeks to 6 months at Mulago national referral hospital in Uganda.

**Methods:**

We conducted a cross-sectional study at Mulago Hospital between September 2016 and March 2017. We enrolled infants born with low birth weight between six weeks and six months whose mothers were available and willing to provide informed consent. Upon obtaining informed consent, we administered a structured questionnaire and performed a physical examination on the participants. Blood was drawn for calcium, phosphorus and vitamin D estimation. We measured serum 25 hydroxy vitamin D (25(OH)D) using the electrochemiluminescence method. Vitamin D deficiency and insufficiency were defined as (25(OH)D) < 20ng/ml and from 20ng/ml to <30 ng/ml respectively. To determine factors associated with vitamin D deficiency, we fit multivariable logistic regression models with exposure factors determined a priori. Data were analysed using Stata version 14.

**Results:**

We enrolled 297 participants, 49.2% (167/297) of whom were males. The median infant age was nine weeks (interquartile range 7–13). All infants had less than one hour of sunlight exposure and over 90.6% (269/297) had received multivitamin supplements containing vitamin D. The prevalence of vitamin D deficiency was 12.1% (36/297): 95% CI (8.9%-16.4%). The prevalence of vitamin D insufficiency was 19.9% (59/297): 95% CI (15.7%-24.8%). Boys had higher odds of vitamin D deficiency compared to girls [adjusted odds ratio 2.8: 95% CI 1.3–6.1].

**Conclusion:**

Vitamin D deficiency was 12.1% among low-birth-weight infants in Uganda although almost all of them had received multivitamin supplements containing vitamin D. We recommend that more studies are done in low-birth-weight infants to assess the risk factors for vitamin D in these population in Uganda.

## Introduction

Vitamin D is a pro-hormone [1] with a vital function of bone mineralization. Furthermore, Vitamin D is a potent immunomodulator that influences the functioning of macrophages and monocytes and participates in the formation of cathelicidin, a peptide capable of destroying several infections including mycobacterium tuberculosis [2, 3]. In infants, vitamin D leads to long term benefits such as improved growth, reduced risk of respiratory infections and broncho asthma [4–6]. In low birth weight (LBW) babies, vitamin D improves bone growth [7].

In humans, the main source of vitamin D is sunshine but in infants, dietary intake and maternal vitamin D status are the main source [8]. Vitamin D deficiency is a widespread problem in the world. In Europe up to 18% of the population is estimated to have vitamin D deficiency while in Canada, it is as high as 37% [9, 10]. Despite the abundance of sunshine, Africa’s prevalence of vitamin D deficiency is as high as 59% in some countries [11].

The prevalence of Vitamin D deficiency in preterm infants is high in several parts of the world. In the USA, the prevalence of vitamin D deficiency or insufficiency among preterm babies is 80% [12]. In East Africa, the prevalence of vitamin D deficiency in infants ranges from 30% to 76% [13–15]. In Uganda, studies have shown that high levels of vitamin D deficiency occur in children with severe malnutrition [16] and those with severe malaria [16, 17].

Preterm and low birth weight infants are a special group because they are at a high risk of metabolic bone disease because of their low phosphorus and calcium stores. Optimal intake of vitamin D is essential to reduce the risk of poor bone mineral deposition and growth. In Uganda, we do not routinely supplement LBW infants with the recommended doses of vitamin D or phosphorus and the breastfeeding mothers are not supplemented with vitamin D routinely. Furthermore, LBW infants are restricted from exposure to the cold thus many of these infants hardly get sunshine exposure in the early months of life. Uganda has a high burden of preterm births [18] and these have a higher risk of death in the neonatal period compared to their normal birth weight counter-parts. Since vitamin D has a role in the immune potency and growth of the infants, determining the burden of vitamin D will assist in addressing its effects and developing guidelines to address vitamin D deficiency. We therefore aimed to determine the prevalence and factors associated with Vitamin D deficiency among LBW infants in a national referral hospital in Uganda.

## Methods

### Study design

We carried out a cross-sectional study at Mulago Hospital. Our study was nested in a bigger study designed to determine the prevalence and factors associated with metabolic bone disease among preterm infants.

### Study setting

The study was carried out at the Mulago Hospital LBW/preterm outpatient clinic. Mulago Hospital is located in Kampala the capital city of Uganda. It is the national referral hospital and teaching hospital for Makerere University Kampala. Uganda is located at the equator and sunshine is abundant throughout the year. The Mulago Hospital neonatal unit commonly called special care unit (SCU) receives over 2000 preterm and low-birth-weight new born admissions annually. These babies are discharged home when they are stable and followed up for growth and any other medical concerns in the LBW/preterm clinic. Children are not routinely screened for vitamin D deficiency but receive routine multivitamin and iron supplementation. The total daily vitamin D from the commonest multivitamins supplement is 100–200 IU of vitamin D. No routine parenteral nutrition or phosphorus supplementation is done even for the extreme low birth weight babies. The study was carried out between the months of September 2016 and March 2017. This season comprises both the wet and dry season. Many children born preterm in our setting are heavily swaddled up even when they are brought out of the houses on warm days because of the fear of hypothermia.

### Study participants

All infants aged six weeks to six months who attended the Mulago Hospital preterm/low birth weight clinic whose birth weights were less than 2.5 kg were included in the study. Those whose biological mothers were not able to give informed consent were excluded from the study. Infants who were known or suspected to have a chronic illnesses like renal and liver disease from medical history or physical examination were also excluded from the study. If participants were twins, we only included the first twin.

### Ethics

Institutional approval was sought from the Makerere University School of Medicine Research and Ethics Committee and written informed consent was sought from the parents of the infants before enrolment. Those who had vitamin D deficiency or rickets were treated.

### Study procedure/data collection

We consecutively enrolled the infants attending the preterm clinic until the sample size for the primary study was attained. The primary study was to determine the prevalence of metabolic bone disease among preterm infants attending the preterm/ low birth weight clinic at Mulago National Referral Hospital. Data were collected by the Principal Investigator (PI) and trained research assistants using a pretested Case Report Form (CRF). The children were screened for eligibility and consented by the research assistant or the PI. An interviewer-administered CRF was filled and variables picked included; antenatal, natal and postnatal history of the mother and infant. The CRF also captured details of nutrition of the child and how often the child is exposed to sunlight per day. We also conducted a detailed physical examination for each child. The routine medications in the preterm clinic such as multivitamin drops and iron supplements were prescribed. The case report form was developed by the principal investigator and reviewed by experienced paediatricians (co-investigators) one of whom is a paediatric endocrinologist for content validity. The questions included in the CRF were standard clinical questions such as age of the child, sex of the child, birth weight, serum vitamin D level, radiographic evidence of rickets, calcium level, Harrison’s sulcus, maternal age, maternal education, maternal HIV status and breastfeeding status.

Blood was drawn from the peripheral veins for biochemical tests: Calcium, Phosphorus and 25-Hydroxy-vitamin D (25(OH) D). Vitamin D level was determined using an immunoassay technique-the Electrochemiluminescence (Elecsys) vitamin D assay. This technique measures the vitamin D concentrations in the range of 4–100 ng/ml. Vitamin D level between 20ng/ml (3ommol/L) to less than 30ng/ml(75mmol/L) was classified as insufficiency while levels below 20ng/ml (30mmol/L) was classified as deficiency [19]. Calcium and phosphorus were measured using COBAS 6000 chemistry analyser. Phosphorus levels below 1.3mmol/L were considered low; calcium values less than 2.3mmol/L were considered to be low.

All Children had wrist radiographs done to assess for radiographic evidence of rickets. Radiographs were read by an experienced radiologist who was blinded to the laboratory results.

### Sample size and data analysis

Primary sample size calculation was not done for this study because it was limited by parent study that enrolled 351 participants. For this study, 297 participants were enrolled. This sample size results in an absolute precision of 1.6% to 5.7%, i.e. the difference between the point estimate and the 95% confidence interval (CI) for prevalence values ranging from 2% to 50%.

Data collected was entered and cleaned using EpiData 3.1 and imported to Stata version 14 for analysis. The prevalence of Vitamin D deficiency or insufficiency was expressed as proportion of the total number of infants recruited into the study. We used Chi-squared (X^2^) test and Fisher’s exact test to determine the association between the infant’s characteristics and maternal clinical and sociodemographic characteristics associated with vitamin D deficiency. To determine factors associated with vitamin D deficiency, we fit an exploratory multivariable logistic regression model with exposure factors determined a priori during literature review [8, 20–22]. The factors that were selected for analysis were child’s age in weeks, child’s sex, birth weight, maternal age, maternal education level, mother’s HIV status, and complications during pregnancy, exclusive breastfeeding and vitamin D supplementation of the child [8, 20–22]. Data were analysed using Stata version 14.0. Upon completion of the exploratory analysis, we explored the association between levels of birth weight (categorized as low birth weight (2.0<2.5kg)), very low birth weight (10–1.9kg), extreme low birth weight(<1.0kg) and vitamin D deficiency (an a posteri analysis) basing on the fact that it was the only variable that showed a meaningful effect measure (and precision) and could be intervened upon (unlike child sex and maternal age). We drew a directed acyclical graph (S1 Appendix) [23], to identify factors that could be controlled for to assess the association between levels of low birth weight and vitamin D deficiency.

## Results

### Characteristics of the participants

**Fig 1:**
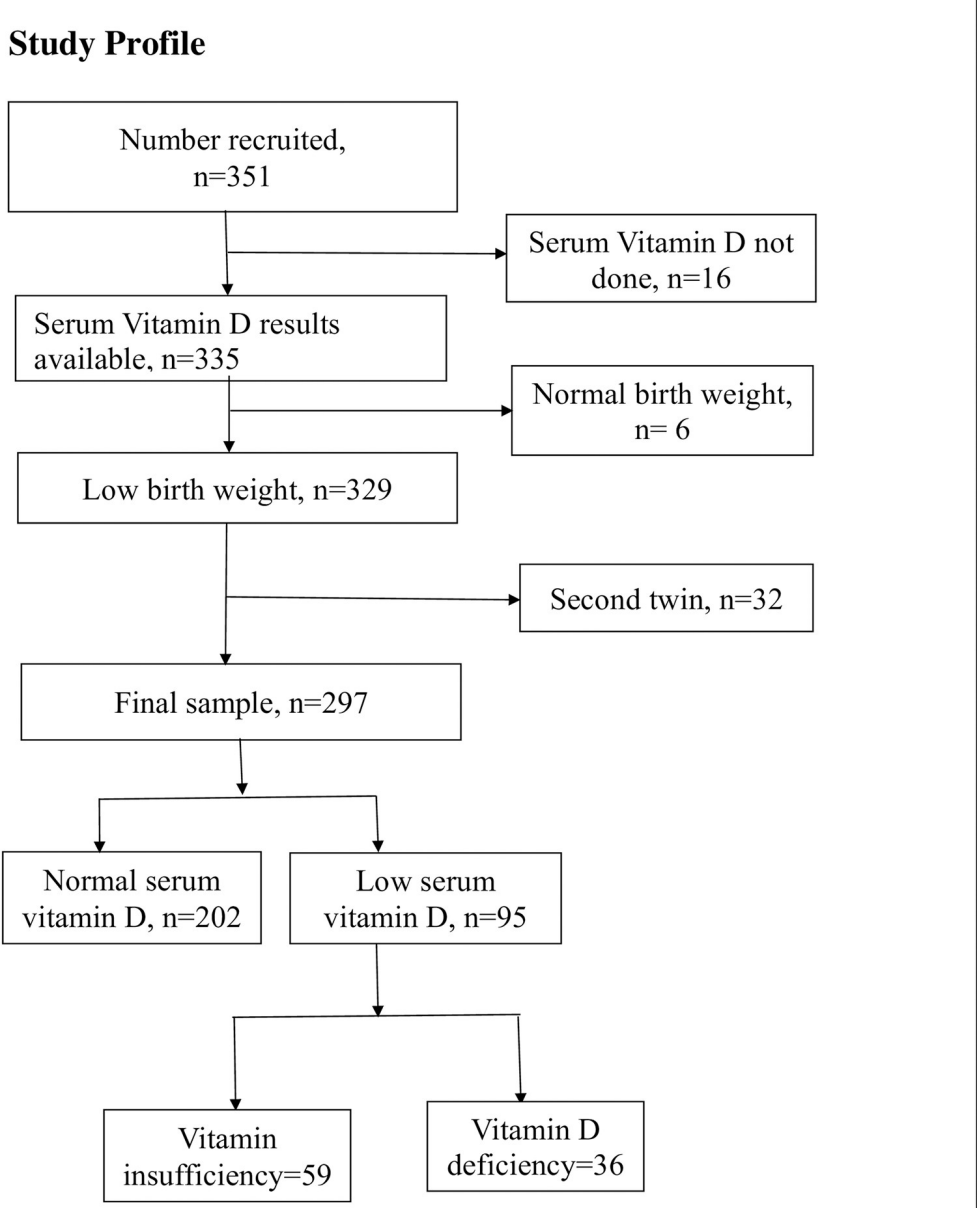
The profile of the study participants.

We included 297 participants in the analysis for this study as shown in the study profile (Fig 1). Of these, 49.2% (167/297) were males. The median infant age was nine weeks (IQR 7–13). The mean gestational age was 31.4weeks (SD 2.88). The mean birth weight was 1573g (SD 366.4) with more than half, (59.6%, 177/297) of the participants having LBW. All infants had less than one hour of sunlight exposure and 90.6% (269/297) had received multivitamin supplements containing vitamin D.

Radiographic evidence of rickets was found in 7.1% (21/297) of the participants. Among the participants, 5.7% (17/297) had low serum phosphorus and 15.2%) (45/297) had low serum calcium. Almost 10% (27/297) of the participants were adolescent mothers and 9.4% 28/297) of the participants were HIV infected. The rest of the characteristics are shown in Table 1.

**Table 1:** Baseline characteristics of the infants and their mothers.

Variable	Frequency (n = 297)	Percentage
**Age (weeks)**		
6 to 12	206	69.4
13–20	62	20.9
21–26	29	9.8
**Sex**		
Male	146	49.2
Female	151	50.8
**Birth weight**		
Low Birth Weight ((2.0–2.5 kg)	177	59.6
Very Low Birth Weight (1.0–1.9 kg)	114	38.4
Extremely Low Birth Weight (<1.0 kg)	6	2.0
**Received vitamin D**		
No	27	9.1
Yes	269	90.9
**Radiographic evidence of rickets**		
Yes	21	7.1
No	276	92.9
**Phosphorus**		
Normal	280	94.3
Hypophosphataemia	17	5.7
**Calcium**		
Normal	252	84.8
Hypocalcaemia	45	15.2
**Harrison’s sulcus**		
No	286	96.3
Yes	11	3.7
**Maternal age (years)**		
19 and below	27	9.1
20–30	202	68.0
>30	68	22.9
**Maternal level of education**		
None	12	4.0
Primary	70	23.6
Secondary	163	54.9
Tertiary	52	17.5
**Mother’s HIV status**		
Negative	269	90.6
Positive	28	9.4
**Maternal complication during pregnancy**		
No	170	57.2
Yes	127	42.8
**Type of complications during pregnancy**		
Febrile illness	41	32.3
Hypertensive disease in pregnancy	47	37.0
Others	39	30.7
**Infant still exclusively breast feeding**		
No	63	21.2
Yes	234	78.8

### Prevalence of vitamin D deficiency

Overall, 12.1% (95% CI 8.85–16.38, n = 36) of the infants had vitamin D deficiency and 32.0% (95% CI 26.90–37.54, n = 95) had vitamin D insufficiency. All children spent less than one hour under sunlight exposure.

### Factors associated with vitamin D deficiency

At exploratory multivariable analysis, boys had higher odds of vitamin D deficiency compared to girls [AOR 2.8: 95% CI 1.3–6.1] (Table 2). The association between levels of low birth weight and vitamin D deficiency was too imprecise to draw conclusion both in the exploratory model (Table 2) and a posteri analysis (S2 Appendix).

**Table 2:** Bivariable and multivariable exploratory logistic regression analysis for the factors associated with vitamin D deficiency.

Age (weeks)	Unadjusted OR (95%CI)	P value	Adjusted Odds Ratio (95% CI)	P value
6 to 12	1		1	
13–20	0.61 (0.22–1.65)	0.320	0.69 [0.24–1.98]	0.485
21–26	1.44 (0.51–4.11)	0.490	2.42 [0.70–8.36]	0.161
**Sex**				
Boys	2.63 (1.24–5.57)	0.010	2.79 [1.28–6.12]	0.010
Girls	1		1	
**Birth weight**				
Low Birth Weight	1		1	
Very Low birth weight	0.72 (0.33–1.53)	0.387	0.64 [0.29–1.43]	0.275
Extremely Low Birth Weight	3.34 (0.58–19.32)	0.177	5.27 [0.75–37.27]	0.096
**Received vitamin D**				
No	1		1	
Yes	0.78 (0.25–2.39)	0.659	1.12 [0.33–3.81]	0.857
**Maternal age**				
19 and below	1		1	
20–30	1.69 (0.375–7.57)	0.50	2.16 [0.45–10.32]	0.336
>30	2.155 (0.38–7.57)	0.344	3.64 [0.65–20.44]	0.143
**Level of Education**				
None	1		1	
Primary	1.83 (0.21–15.80)	0.58	1.21 [0.12–2.68]	0.875
Secondary	1.45 (0.18–11.17)	0.73	1.39 [0.13–14.71]	0.785
Tertiary	1.43 (0.16–13.17)	0.75	1.26 [0.11–14.98]	0.852
**Mother’s HIV status**				
Negative	1		1	
Positive	1.2 (0.40–3.79)	0.71	1.06 [0.32–3.55]	0.928
**Maternal complication during pregnancy**				
No	1		1	
Yes	0.63 (0.30–1.32)	0.23	0.55 [0.25–1.20]	0.131
**Infant still exclusively breast feeding**				
No	1		1	
Yes	1.4 (0.55–3.52)	0.48	1.99 [0.69–5.68]	0.201

### Laboratory and radiological investigations

Infants with vitamin D deficiency had slightly lower blood calcium levels [median (IQR): 2.37 (2.28–2.48)] compared to infants without vitamin D deficiency [median (IQR): 2.53 (2.3–2.65)]. Infants with vitamin D deficiency had slightly lower blood phosphorous levels [median (IQR): 2.13 (1.88–2.89)] compared to infants without vitamin D deficiency [median (IQR): 2.2 (1.81–2.78)]. Only 2/36 (5.6%) of infants with vitamin D deficiency had radiological rickets, compared to 19/261 (7.3%) of infants without vitamin D deficiency.

## Discussion

In this study, we found that the prevalence of vitamin D deficiency was (12.1%) despite the almost universal supplementation with vitamin D. The amount of vitamin D (100–200 IU) in the supplements that these infants were taking may be suboptimal for this population with limited outdoor exposure. Furthermore, although adherence to vitamin supplementation was not assessed, it is possible that many of the participants had poor adherence.

We did not find any published data about prevalence of vitamin D deficiency among healthy Ugandan infants. This prevalence is similar to findings in a cross-sectional study by Nabeta et al in Uganda who found vitamin D insufficiency in 29.5% and deficiency in 12% of children with protein energy malnutrition [16]. In the same study, it was also found that vitamin D insufficiency and deficiency was 19.5% and 14% respectively among children who did not have protein-energy malnutrition in Uganda [16]. However, our findings are much lower than findings by Cusick et al who found the prevalence of vitamin D insufficiency at 80% and deficiency at 30% among children with severe malaria in Uganda [17]. The difference in findings could be because most of the infants in this study had multivitamin supplementation but also children with severe malaria are critically ill and are more likely to have much less sunshine exposure. Additionally, the sample size in Cusick et al was so small and thus the high prevalence of Vitamin D deficiency and vitamin D insufficiency. A study done by Urio et al found higher prevalence of Vitamin D deficiency of 22% among infants attending a reproductive and child health clinic in Arusha, Tanzania [24]. The prevalence of Vitamin D deficiency in our study is also lower than the findings of Said et al. who found that 23.4% of exclusively breast fed infants had vitamin D levels below 20ng/ml in a tertiary healthcare facility in Nairobi, Kenya [25]. The differences between our study findings and these findings may be because the participants in our study were on vitamin D supplementation. A study of preterm babies receiving vitamin D supplementation in the United States of America found much higher values than found in this study possibly because, being in the tropics, the infants in this study may be receiving a better supply of Vitamin D through breast feeding from their mothers who are exposed to sunshine more frequently [12].

In our study, males were found to be more likely to have vitamin D deficiency than female infants possibly because of low vitamin D levels in the mothers of the male infants. Several studies have shown that mothers who give birth to male infants have lower vitamin D levels than those who give birth to female infants [20, 26]. In this study however, we did not measure serum vitamin D levels in the mothers. The exact reason for the difference in vitamin D levels between boys and girls is not yet known and could be an area for future research. There are studies that have shown that girls were more likely to have vitamin D insufficiency. This association is thought to be due to the less time the girls spend outdoors and the concealing clothing they wear which reduces exposure to sunshine [27, 28]. This is true for the older children. In this study all the infants had limited exposure outdoors.

Infants with vitamin D deficiency had low serum calcium and low serum phosphorus. Vitamin D is vital in the absorption of calcium and phosphorus from the intestines. Vitamin D improves the absorption of calcium and phosphorus by 30–40% and 80%, respectively [19]. Vitamin D deficiency may therefore lead to low serum calcium and phosphorus.

Only a small proportion of infants who had radiologic rickets also had vitamin D deficiency because the radiologic findings may not be related to vitamin D deficiency but may be due metabolic bone disease (MBD) of prematurity. Most of the calcium and phosphorus accretion into the foetus from the placenta occurs in the third trimester of gestation [29]. Therefore, preterm infants are born before they acquire adequate calcium and phosphorus stores [30]. This eventually leads to poor bone mineral density in these infants. This condition is called metabolic bone disease.

This study had some limitations. We did not assess the adherence to multivitamin that the infants were taking daily. We did not test for PTH level; this would have helped us assess the association between the PTH and serum vitamin D, phosphorus and calcium.

## Conclusion

Vitamin D deficiency prevalence was 12.1% among children with low-birth-weight infants in Uganda although almost all of them had received multivitamin supplements containing vitamin D. Boys had higher odds of vitamin D deficiency compared to girls. We recommend that more studies are done in preterm babies to assess the risk factors for vitamin D in these population in Uganda. Vitamin D supplementation for mothers of preterm infants should be considered to reduce vitamin D deficiency risk among their babies.

## Supporting information

S1 Appendix Directed acyclical graph (DAG) for causal association between birthweight and vitamin D deficiency. (TIF)

S2 Appendix Table for aposteri logistic regression analysis of association between birth weight and vitamin D deficiency. (DOCX)

S1 Data (DOCX)

S1 Dataset (DTA)
