# Supplementary figures and images for: Vitamin D deficiency in low-birth-weight infants in Uganda; a cross sectional study
Source: PLoS One. 2022 Nov 11;17(11):e0276182. doi: 10.1371/journal.pone.0276182 (PMC9651562; doi:10.1371/journal.pone.0276182)

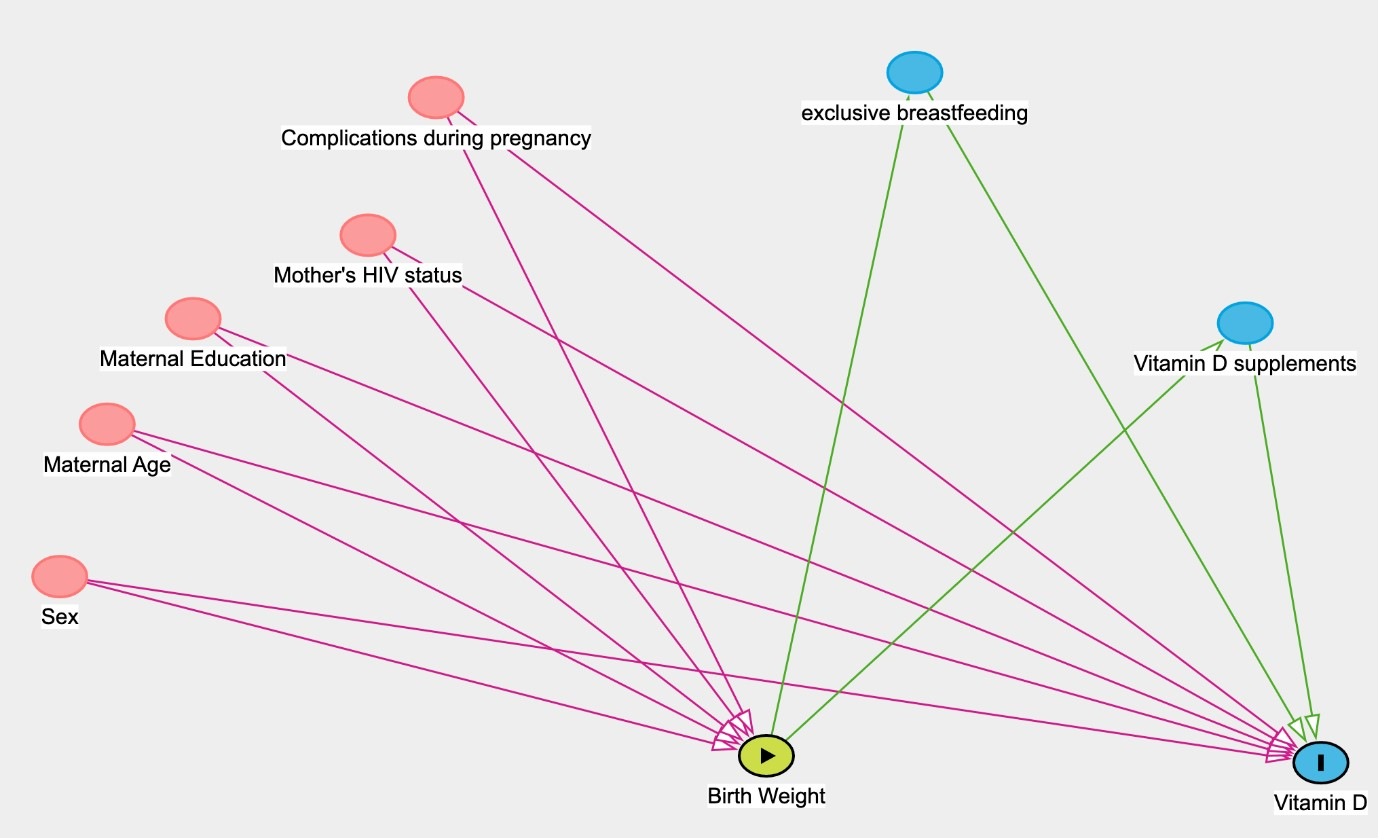

Supplement: S1 Appendix — (TIF) [file pone.0276182.s001.tif]
