# Supplementary material for: Vitamin D deficiency in low-birth-weight infants in Uganda; a cross sectional study
Source: PLoS One. 2022 Nov 11;17(11):e0276182. doi: 10.1371/journal.pone.0276182 (PMC9651562; doi:10.1371/journal.pone.0276182)
Supplement: S2 Appendix — (DOCX) [file pone.0276182.s002.docx]

**Table 3: Multivariable logistic regression analysis for the association between birth weight and vitamin D deficiency**.

| **Age (weeks)** | Unadjusted OR (95%CI) | P value | Adjusted Odds Ratio (95% CI) | P value |
| --- | --- | --- | --- | --- |
| **Birth weight** |  |  |  |  |
| Low Birth Weight | **1** |  | **1** |  |
| Very Low birth weight | **0.72 (0.33**–**1.53)** | **0.387** | **0.64 [0.29**–**1.40]** | **0.266** |
| Extremely Low Birth Weight | **3.34 (0.58**–**19.32)** | **0.177** | **4.43 [0.69**–**28.62]** | **0.118** |
| **Sex** |  |  |  |  |
| Male | 2.63 (1.24–5.57) | 0.010 | 2.62 [1.22–5.65] | 0.014 |
| Female | 1 |  | 1 |  |
| **Maternal age** |  |  |  |  |
| 19 and below | 1 |  | 1 |  |
| 20-30 | 1.69 (0.375–7.57) | 0.50 | 2.01 [0.43–9.52] | 0.378 |
| >30 | 2.155 (0.38–7.57) | 0.344 | 3.00 [0.55–16.35] | 0.205 |
| **Level of Education** |  |  |  |  |
| None | 1 |  | 1 |  |
| Primary | 1.83 (0.21–15.80) | 0.58 | 1.32 [0.14 –12.71] | 0.808 |
| Secondary | 1.45 (0.18–11.17) | 0.73 | 1.33 [0.14 – 12.60] | 0.802 |
| Tertiary | 1.43 (0.16–13.17) | 0.75 | 1.32 [0.12 –14.01] | 0.817 |
| **Mother’s HIV status** |  |  |  |  |
| Negative | 1 |  | 1 |  |
| Positive | 1.2 (0.40–3.79) | 0.71 | 1.16 [0.36–3.81] | 0.801 |
| **Maternal complication during pregnancy** |  |  |  |  |
| No | 1 |  | 1 |  |
| Yes | 0.63 (0.30–1.32) | 0.23 | 0.58 [0.27–1.25] | 0.167 |


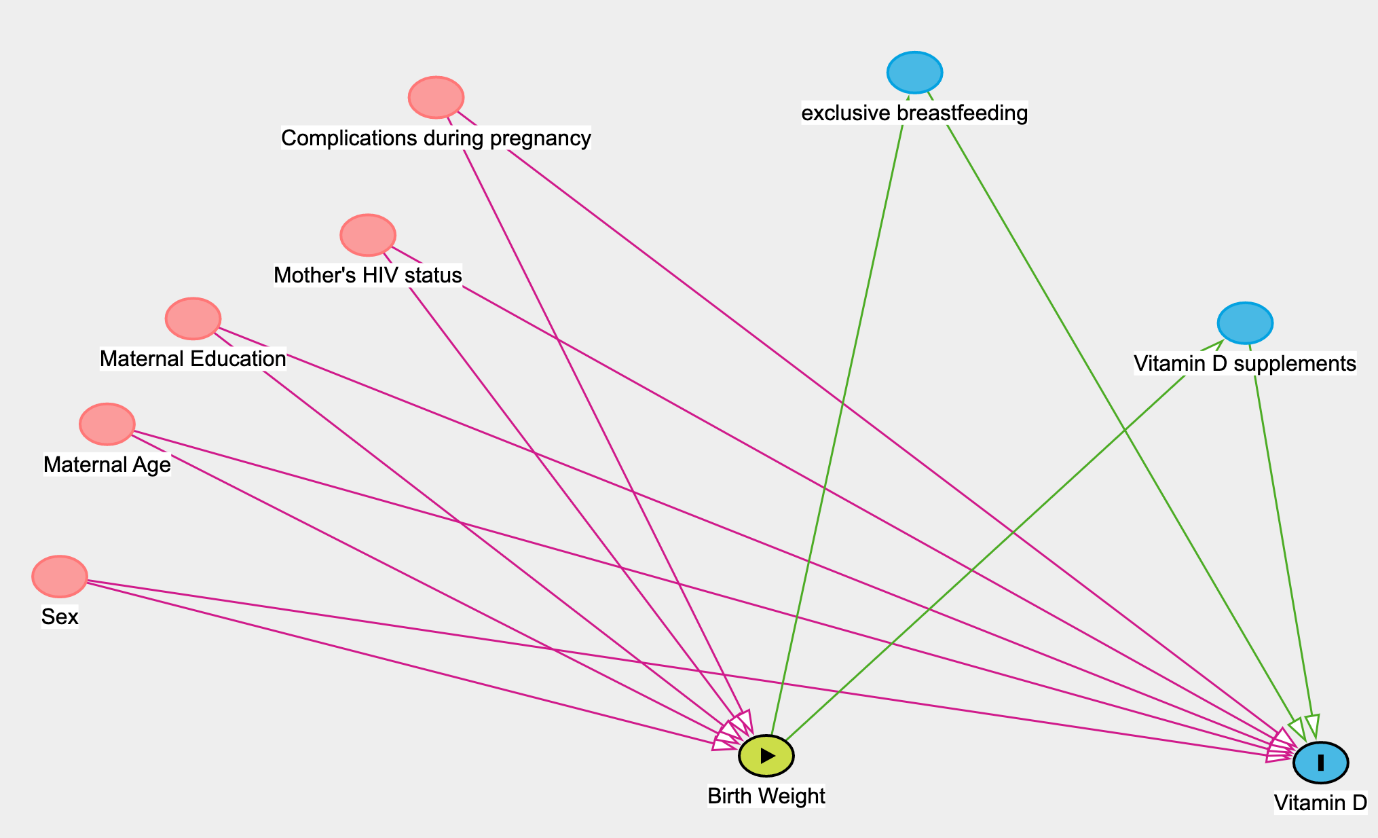


Figure 2: Directed acyclic graph showing variables to adjust for in assessing association between birth weight and vitamin D.
