# Supplementary material for: Vitamin D deficiency in low-birth-weight infants in Uganda; a cross sectional study
Source: PLoS One. 2022 Nov 11;17(11):e0276182. doi: 10.1371/journal.pone.0276182 (PMC9651562; doi:10.1371/journal.pone.0276182)
Supplement: S1 Data — (DOCX) [file pone.0276182.s003.docx]

**APPENDIX 1: DATA COLLECTION TOOL**

PREVALENCE OF METABOLIC BONE DIEASE AND ASSOCIATED FACTORS AMONG CHILDREN ATTENDING PRETERM CLINIC IN MULAGO HOSPITAL

**Name: ...................................................................... ID (Study No.)…………………………**

| **No.** | **Questions** | | **Response** |
| --- | --- | --- | --- |
| 101 | Date of recruitment | | …………………… |
| 102 | Participant Age | | Months……….Weeks……. |
| 103 | Date of birth | |  |
| 104 | Gender | | 1. Male 2. Female |
| 105 | Mother’s last normal menstrual period | |  |
| 106 | Gestational age at birth | |  |
| 107 | Birth weight (kg) | |  |
| 108 | Current weight (kg) | |  |
| 109 | Birth Order | |  |
| 110 | Multiple gestation? | | 1. Yes 2. No |
|  | **Demographic Data** | |  |
| 111 | Mother’s Age | | ..............yrs |
| 112 | Mother’s marital status | | 1. Married/cohabiting 2. Single/separated/widowed |
| 113 | Mother’s education status | | 1. None 2. Primary 3. Secondary 4. Tertiary |
| 114 | Mother’s occupation | |  |
|  | **Pregnancy Related History** | |  |
| 115 | Did you smoke during pregnancy | | 1. Yes 2. No |
| 116 | Did you take alcohol during this pregnancy? | | 1. Yes 2. No |
| 117 | Maternal complications during pregnancy? | | 1. Yes 2. No |
| 118 | Nature of the complication | | 1. High blood pressure 2. Febrile illness 3. Other(specify)…………….. |
| 119 | Did you take calcium supplements during pregnancy? | | 1. Yes 2. No |
| 120 | Did you have muscle pools/crumps during pregnancy | | Yes No |
| 121 | Mother’s HIV status | | 1. Positive 2. Negative 3. unknown |
| 122 | Were you taking ARVs during pregnancy or after birth? | | 1. Yes 2. No |
| 123 | If yes, what regimen | | 1.zidovudine 2 Lamivudine 3 tenofovir 4 Efavirenz 5 nevirapine 6-others(specify..............................) |
| 124 | Baby’s HIV status | | - 1. Positive 2. Negative 3 unknown |
| 125 | If HIV positive, is the child on ARVS | | 1 Yes 2. No 3. Not applicable |
| 126 | What was the cause of the preterm labour? | | 1. Febrile illness 2. Urinary tract infection 3. Doctor’s intervention to save mother’s life 4. Spontaneous 5. Trauma 6. Other(specify)...................................... |
|  | **Postnatal History** | |  |
| 127 | Did the child cry immediately after birth? | | 1. Yes 2. No |
| 128 | Was the child put on Oxygen after birth? | | 1. Yes 2. No |
| 129 | If yes to no. 125 above, how long after birth? | | …….weeks………days |
| 130 | Did the child’s body turn yellow after birth | | 1. Yes 2. No |
| 131 | Was the child put on any of the following to treat yellow colour on the body | | 1 Phototherapy 2 exchange transfusion |
| 132 | Did the child develop Fever in the first one month of life | | 1. Yes 2. No |
| 133 | Has child ever been admitted to Special Care Unit? | | 1. Yes 2. No |
| 134 | If yes to no.133, for how long | | ……hrs……..days……….weeks |
|  | **Feeding History** | |  |
| 135 | What was the Age of initiation of oral feeds? | | ……hrs……..days……….weeks |
| 136 | What was the Age of full feeds through the mouth? | | ……hrs……..days……….weeks |
| 137 | Was the child ever fed through a feeding tube | | 1. Yes 2. No |
| 138 | If yes to no.135, for how long? | | ……hrs……..days……….weeks……..months |
| 139 | Did the child have convulsions after birth | | 1. Yes 2. No |
| 140 | Has the child been on any of the following medications: | | \| Drug Name \| taken \| \| Age of starting \| Duration of  intake \| \| --- \| --- \| --- \| --- \| --- \| \|  \| Yes \| No \|  \|  \| \| Diuretics(furosemide) \|  \|  \|  \|  \| \| Phenytoin \|  \|  \|  \|  \| \| Phenobarbitone \|  \|  \|  \|  \| \| aminophyline \|  \|  \|  \|  \| \| Steroids(prednisolone, hydrocortisone) \|  \|  \|  \|  \| |
|  | **Nutrition history:** | |  |
| 139 | Exclusively breast fed for, | | ……..weeks………months |
| 140 | Does the child still breast feed? | | 1. Yes 2. No |
| 141 | If no to no.140 above, duration of breast feeding | | ……..weeks………months |
| 142 | Reason for not exclusively breast feeding for six months | |  |
| 138 | \| Feed \| taken \| \| Still taking \| \| Age of starting \| Age of starting \| dilution \| \| --- \| --- \| --- \| --- \| --- \| --- \| --- \| --- \| \|  \| Yes \| No \| Yes \| No \|  \|  \|  \| \| Formula-term \|  \|  \|  \|  \|  \|  \|  \| \| Formula-preterm \|  \|  \|  \|  \|  \|  \|  \| \| Cow’s milk \|  \|  \|  \|  \|  \|  \|  \| \| Porridge; \|  \|  \|  \|  \|  \|  \|  \| \| 1………………. \|  \|  \|  \|  \|  \|  \|  \| \| 2………………. \|  \|  \|  \|  \|  \|  \|  \| \| 3………………. \|  \|  \|  \|  \|  \|  \|  \| | | |
| 140 | For how long did the child receive the following supplements? | |  |
| 141 | \|  \| supplements \|  \| \| \| \| \| \| \| amount \| \| --- \| --- \| --- \| --- \| --- \| --- \| --- \| --- \| --- \| --- \| \|  \|  \|  \| \| \| \| \| \| \|  \| \|  \|  \| Yes \| no \| duration \|  \|  \| Doses missed \| Last dose \|  \| \| a \| haemorforte \|  \|  \|  \|  \|  \|  \|  \|  \| \| d. \| Grovit \|  \|  \|  \|  \|  \|  \|  \|  \| \| e. \| Vitamin D(specify name of syrup)……………… \|  \|  \|  \|  \|  \|  \|  \|  \| \| f. \|  \|  \|  \|  \|  \|  \|  \|  \|  \| \| g. \|  \|  \|  \|  \|  \|  \|  \|  \|  \| | | |
| 142 | How often is the child exposed to sunlight per day: | | 1. < 1 hr 2. 2hrs -3hrs 3. 4hrs-5 hrs 4. > 5hrs |
| 143 | Has your child been diagnosed of any chronic illness | | 1. Yes 2. No |
| 144 | If yes which one | |  |
| 145 | History of previous admission | | 1. Yes 2. No |
| 146 | If yes to above question, complete this table | \|  \| Age at admission \| Duration of admission \| Reason for admission \| \| --- \| --- \| --- \| --- \| \| a \|  \|  \|  \| \| b \|  \|  \|  \| \| c \|  \|  \|  \| \| d \|  \|  \|  \| | |
|  |  |  | |
| 147 | Milestones | Record of milestones   \| Miles stone \| attained \| \| Age attained \| \| --- \| --- \| --- \| --- \| \|  \| yes \| no \|  \| \| Social smile \|  \|  \|  \| \| sitting \|  \|  \|  \| \|  \|  \|  \|  \| | |

|  | GENERAL EXAMIANTION |  |
| --- | --- | --- |
| 148 | Oedema 1. Yes 2. No | 149. Wasting 1. Yes 2. No |
| 150. | Pallor 1. Yes 2. No | 151. Bossing 1. Yes 2. No |
| 152 | Craniotabes 1. Yes 2. No | 153. Rachitic rosary 1. Yes 2. No |
| 154 | Harison’s sulcus 1.Yes 2. No | 155. Widened wrists 1. Yes 2. No |
| 156 | Chest deformities 1. Yes 2. No | 157. Bowing of Limbs 1. Yes 2.No |
| 158 | Clinical evidence of fractures  1. Yes 2. No | 159. Anterior fontanelle size: ……. By …………cm |
| 160 | Suture diastesis 1. Yes 2. No |  |
|  |  |  |
| RESULTS OF INVESTIGATION | | |
|  |  |  |
| Vitamin D |  |  |
| Calcium |  |  |
| ALP |  |  |
| Phosphorus |  |  |
| Radiograph | 1. Early rickets 2. Established rickets 3. Ostepenia 4. Fractures |  |
